# Meta-analysis of Shugan Jieyu Capsule for depression in patients with coronary heart disease

**DOI:** 10.1097/MD.0000000000034685

**Published:** 2023-08-25

**Authors:** Zhen Liu, Chunhua Gu, Jian Lei

**Affiliations:** a Department of Internal Medicine, Bao ‘an Hospital of Traditional Chinese Medicine, Shenzhen, Guangdong, China; b Emergency Department, Baoan District People’s Hospital, Shenzhen, Guangdong, China; c Department of Cardiology, Wuhan First Hospital, Wuhan Hospital of Integrated Traditional Chinese and Western Medicine, Wuhan, Hubei, China.

**Keywords:** cardiovascular disease, coronary heart disease, meta-analysis, Shugan Jieyu capsule, trial sequential analysis

## Abstract

**Introduction:**

Shugan Jieyu Capsule (SGJYC) has been prescribed to treat primary and secondary depression; however, whether it can benefit depression of patients with coronary heart disease (CHD) remains controversial. This meta-analysis aimed to evaluate the efficacy and safety of SGJYC in treating depression in patients with CHD.

**Patient concerns:**

A total of 644 CHD patients with depression were selected from China National Knowledge Infrastructure, Wanfang, China Biomedical Database, MEDLINE, the Cochrane library from their inceptions until June, 2021.

**Diagnosis:**

All patients with CHD or coronary artery disease were confirmed to suffer from depression based on recognized criteria.

**Intervention:**

Patients were assigned randomly to receive SGJYC-based regimens or conventional antidepressants alone.

**Outcomes:**

Meta-analysis of 6 studies showed that antidepressants (MD, 2.12; 95% confidence interval [CI], 0.73~3.50) or sertraline (MD, 2.15; 95%CI, 0.61~3.68) significantly alleviated depression level compared to SGJYC; however, SGJYC plus antihypertensive drugs (AHD) (MD, −8.33; 95%CI, −13.90 ~ −2.75) significantly improved depression symptoms compared to AHD. A significant difference in risk of adverse cardiac events (risk ratios [RR], 2.72; 95%CI, 1.07~6.94) between SGJYC and sertraline was detected in patients with simple CHD.

**Conclusions:**

SGJYC has a poor effect on depressive symptoms, and the effect of combination with AHD is better than AHD but its efficacy and cardiac safety are inferior to antidepressants.

## 1. Introduction

Coronary heart disease (CHD) is an ischemic cardiovascular disease,^[1]^ with an increasing prevalence worldwide, especially in elderly populations,^[2]^ and it seriously threatens the physical and mental health of patients.^[3]^ Currently, psychonomatic factors play a important role in the occurrence and development of CHD, and approximately 55.3% of patients with CHD have been detected to experience varying degrees of negative psychological symptoms.^[4]^ It is reported that several psychosocial factors were closely associated with high cardiovascular disease mortality,^[5]^ and depression is regarded as one of the most significant factors relating to CHD events^[6]^ and poor prognosis after acute myocardial infarction (AMI).^[7]^ Anxiety and depression are independent risk factors of CHD, which can increase the risk of stable angina pectoris. Anxiety and depression can also promote stable angina pectoris to develop into unstable angina pectoris and AMI, increase the occurrence of adverse endpoint events, and increase cardiovascular mortality and all-cause mortality.^[8]^ The more severe the symptoms of anxiety and depression, the worse the prognosis, and the higher the incidence of adverse endpoint events. For patients with stable angina pectoris, the ultimate goal of treatment is to improve the quality of life with disease, delay the progress of disease, and extend life expectancy, so it is particularly important to intervene in their anxiety and depression.^[9]^

In addition, depression has also been demonstrated to be the major contributor to a higher incidence of major adverse cardiovascular events and risk of all-cause mortality among patients with CHD who received the percutaneous coronary intervention.^[10]^ The dose-response association between the severity of depression and poor prognosis has also been evidenced.^[11]^ For hypertension patients with anxiety and depression, the combination of Shugan Jieyu Capsule (SGJYC) and antihypertensive treatment can make the antihypertensive effect more significant, and can significantly relieve the anxiety and depression symptoms of patients.^[12]^

Although the underlying mechanisms contributing to adverse cardiovascular outcomes among patients with CHD complicated with depression are yet to be fully elucidated,^[13]^ the concurrent use of antidepressants and cardiovascular agents has been proven to better the prognosis and quality of life.^[14]^ However, conventional antidepressants commonly used in clinical practice to treat depression were insufficient and far from ideal.^[15]^ This is because these antidepressants’ administration was associated with numerous side effects and even cardiovascular adverse reactions.^[16]^ These drive an urgent need for identifying novel drugs with fewer side effects.

Chinese patent medicines have been extensively used for treating chronic diseases in China. As a kind of proprietary Chinese medicine, The active ingredients of the SGJYC contained Acanthopanax 1800g and Hypericum perforatum L 1500g.^[17]^ It was strictly prepared according to Chinese pharmacopeia and accurately measured using the high-performance liquid chromatography method. The modern pharmacological study has revealed the antidepressant effect of SGJYC,^[18]^ and China Food and Drug Administration also legally approved SGJYC for clinical prescription in 2008.^[19]^ Compared with the CNS-orientated and single target characteristics of conventional antidepressants, the holistic, multi target and multi-objective characteristics of SGJYC are more consistent with the concept of systematic treatment of depression,^[20]^ providing a better choice for the treatment of depression.^[21]^ According to a previous text mining study, SGJYC has been widely used for clinically treating primary and secondary depression in China.^[22]^ A recent meta-analysis revealed a notable therapeutic efficacy of SGJYC in treating adults with post-stroke depression.^[23]^ Furthermore, a resting-state functional magnetic resonance imaging study revealed the neuroimaging mechanism by which SGJYC improves post-stroke depression, indicating that SGJYC may ameliorate post-stroke depression by affecting brain region activity and local synchronization.^[24]^

Considering the anti-depressive effects and fewer side effects of SGJYC, some clinical trials^[25–30]^ have also been conducted to evaluate the therapeutic efficacy and safety of SGJYC for treating depression in patients with CHD. However, these clinical trials reported conflicting results due to the insufficient sample size, significantly confusing clinicians’ decision-making for CHD patients complicated with depression. Therefore, we conducted this meta-analysis to evaluate the anti-depressive effect and safety of SGJYC by combining the results of currently available clinical trials.

## 2. Methods

We performed this meta-analysis in strict accordance with the Cochrane handbook^[31]^ although we did not register the protocol of our meta-analysis on a public platform. We reported the pooled results in accordance with the Preferred Reporting Items for Systematic Reviews and Meta-Analyses statement.^[32,33]^ No ethical approval and informed consent were required because no person was directly involved in this study.

### 2.1. Eligibility criteria

We developed the following inclusion criteria to assist the selection of eligible studies: adult patients were confirmed with CHD or coronary artery disease; the SGJYC-based regimen was used in the research group, and the conventional antidepressants alone was used in the control group; the study contained indicators evaluating comparative anti-depressive effect and safety of SGJYC-based regimen versus conventional antidepressants; and full texts of articles were available in English or Chinese.

We excluded ineligible studies based on the following exclusion criteria: the study was classified as a review, comment, or letter to the editor; duplicate reports of the same publication with less information, and comparisons were made with other interventions.

### 2.2. Search strategy

We performed a systematic search in China National Knowledge Infrastructure, WANFANG data, Chinese Biomedical Literature database (CBM), MEDLINE, EMBASE, and the Cochrane Controlled Register of Trials (CENTRAL) from their inceptions until June, 2021. We did not restrict language in the literature search. We used the following search terms to construct the search strategy: (“coronary diseases” or “coronary heart disease” or “coronary heart diseases” or “myocardial infarctions” or “cardiovascular stroke” or “cardiovascular strokes” or “myocardial infarct” or “myocardial infarcts” or “heart attack” or “heart attacks” or (“coronary disease [mesh]” or “myocardial infarction [mesh]”)) and (anxiety or depression or (anxiety [mesh] or depression [mesh])) and (shuganjieyu or “shugan jieyu” or shugan-jieyu or shu-gan-jie-yu) and (random or (random allocation [mesh] or randomized controlled trial [mesh])) (see Table S1, http://links.lww.com/MD/J503, Supplemental Content, which illustrates the search strategy in each database).

We also manually checked reference lists of all included studies to identify additional studies.^[34]^ We did not consider those conference abstracts without sufficient data. A third reviewer resolved any disagreements between the 2 reviewers.

### 2.3. Study selection

We selected eligible studies according to the following 3 steps. First, we imported all retrieved citations into EndNote (version X7; Thomson Reuters) and automatically detected and removed duplications. Second, we assigned 2 independent reviewers initially to exclude irrelevant citations by checking the titles and abstracts of retrieved citations. Third, the reviewers retrieved the full text of potentially eligible citations for the final evaluation of eligibility. A third reviewer resolved any disagreements between reviewers.

### 2.4. Data extraction

Two independent reviewers extracted the following data from original studies, including the name of the leading author, year of publication, country, disease, sample size, the proportion of female participants, average ages, details of comparisons, duration of treatment, outcomes. We also extracted information to examine the risk of bias. We emailed the corresponding authors to obtain the essential data when necessary. We used the changes in the Hamilton Depression Scale score between the baseline and the end of the intervention to evaluate the therapeutic effect of SGJYC on depression with the method proposed by Cochrane.^[35]^ A third reviewer resolved any disagreements between reviewers.

### 2.5. Primary and secondary outcomes

We defined the anti-depressive effect and cardiac safety as the primary outcome. The anti-depressive effect was assessed using the changes in HAMD score between the baseline and the end of the treatment. Cardiac safety was assessed by using the number of cases that left ventricular ejection fraction reduced more than 5%. Other adverse events that researchers recorded in the studies were defined as the secondary outcomes.

### 2.6. Assessment of risk of bias

The Cochrane risk of bias assessment tool was used for assessing the risk of bias^[36]^ from 7 domains: random sequence generation, allocation concealment, blinding of participants and personnel, blinding of outcome assessment, incomplete outcome data, selective reporting, and other bias. Every domain was labeled with “low,” “high,” or “unclear” risk according to the criteria recommended by the Cochrane Collaboration. The overall quality of the individual study was judged to be high if 7 domains were low risk, moderate if at least 1 of the 7 domains was an unclear risk and no item was high risk, or low if at least 1 of 7 domains was high risk. A third reviewer resolved any disagreements between reviewers.

### 2.7. Statistical analysis

We used the Review Manager software (RevMan, version 5.3.5; Nordic Cochrane Centre, The Cochrane Collaboration, Copenhagen, Denmark) to perform statistical analysis. The changes in HAMD score were expressed using the standard mean difference with a 95% confidence interval (CI), and cardiac safety was expressed using the risk ratios (RR) with a 95% CI. Heterogeneity across eligible studies was evaluated using chi square (Cochrane Q) and I^2^ statistic. We used the random-effects model to calculate all estimates because variations across studies exist. Moreover, we also performed sensitivity analysis by excluding those studies with inconsistent design, comparator, and studies with high risk. We also calculated the pooled estimates based on different statistical methods, including DerSimonian-Laird,^[37]^ Sidik-Jonkman,^[38]^ and Biggerstaff-Tweedie^[39]^ to evaluate the robustness of pooled results. We did not assess publication bias because the number of accumulated eligible studies was not more than 10.^[36]^ For all tests, statistical significance was defined as a *P* < .05.

### 2.8. Trial sequential analysis

Trial sequential analysis is a statistical method that can determine whether the evidence accumulating in the meta-analysis is reliable and conclusive.^[40]^ We performed a trial sequential analysis for our primary outcomes. The required sample size (RIS) was calculated to determine whether the evidence in our meta-analysis is reliable and conclusive based on the observed data and trial sequential monitoring boundaries.^[41]^ Suppose the accumulated number of patients in the meta-analysis exceeds the RIS or the cumulative Z-value curve crosses through the trial sequential monitoring boundary. In that case, the meta-analysis results are likely stable, and no further study is required. Otherwise, it indicates insufficient evidence to draw a definitive conclusion and future research is required.^[40]^

The diversity-adjusted information size and O’Brien–Flemingα-spending boundaries were estimated using a 2-sided 5% type I error and 20% type 2 error rate (indicating a statistical power of 80%), and the mean difference and variance were calculated from those studies with low risk.^[42]^ The heterogeneity correction was based on the model variance. The software TSA version 0.9.5.10 beta was used for these analyses.

## 3. Results

### 3.1. Search of literature

**Figure 1.**
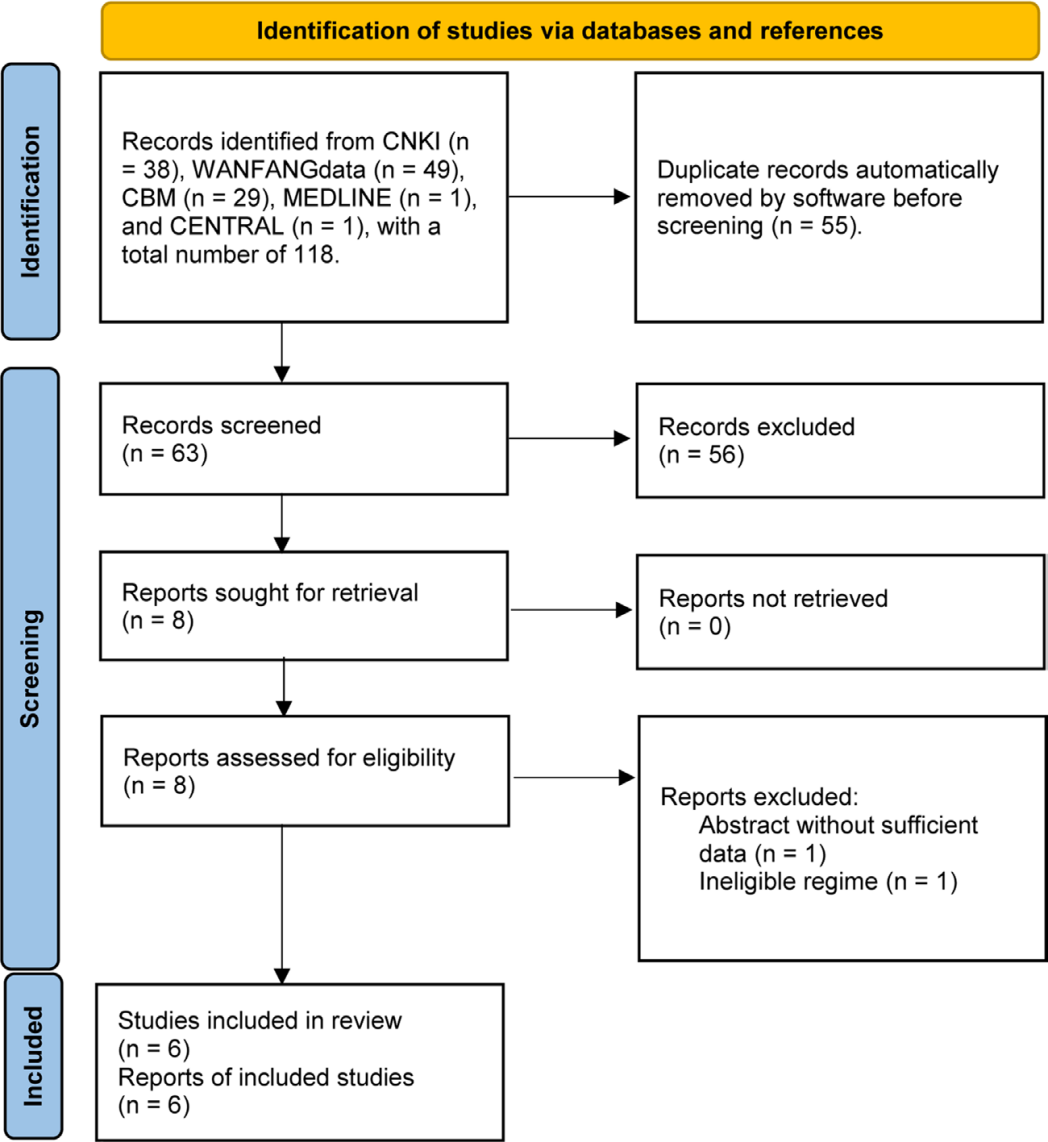
Flow chart of the study retrieval and selection process. CNKI = China national knowledge infrastructure. CBM = Chinese Biomedical Literature database, CENTRAL = the Cochrane Controlled Register of Trial. MEDLINE was searched through PubMed, and CENTRAL was searched through OVID platform.

We initially identified 118 citations from the target databases and retained 63 records for eligibility evaluation after automatically removing duplicate citations. We retrieved the full texts of 8 potentially eligible studies after excluding 56 irrelevant studies based on the title and abstracts screening. Finally, we included 6 eligible studies^[25–30]^ in this meta-analysis after excluding 2 studies due to conference abstract without sufficient information (n = 1) and ineligible regimens (n = 1). The flow chart of study retrieval and selection is displayed in Figure 1.

### 3.2. Characteristics of included studies

We developed Table 1 to summarize the basic characteristics of 6 eligible studies. All studies were performed in China and were published between 2011 and 2021. Three studies^[26,27,30]^ enrolled patients with CHD, 2 studies^[25,28]^ enrolled patients with chronic heart failure, and 1 study^[29]^ enrolled patients with AMI. The sample size of the individual study was between 60 and 155, with a total number of 644 patients (324 patients in the SGJYC group and 320 in the control group). Three studies^[26,27,29]^ prescribed sertraline and 1 study^[25]^ used fluoxetine as control regimen, respectively. Four studies^[25–27,29]^ designed a treatment duration of 12 weeks, another 2 studies^[28,30]^ had an 8-weeks treatment duration. All studies evaluated the comparative effect of SGJYC and comparators based on HAMD score. Three studies^[26,27,29]^ reported cardiac safety after the treatment, and 2 studies^[28,29]^ reported adverse events after treatment.

**Table 1:** Characteristics of included studies (n = 6).

Reference	Condition	Sample size (female)	Average age	Comparison	Treatment duration	Outcomes
Liu et al, 2017	AMI	76 (33) vs 73 (30)	53.4 vs 54.1	SJC vs sertraline	12 wk	HAMD, CA, AEs
Chang et al, 2012	CHF	30 vs 30 (34)	65–80	SJC vs fluoxetine	12 wk	HAMD
Chang et al, 2020	CHD	60 (26) vs 60 (24)	61.5 vs 62.0	SJC vs sertraline	12 wk	HAMD, CA
Chen et al, 2018	CHD	50 (21) vs 50 (22)	61.8 vs 61.5	SJC vs sertraline	12 wk	HAMD, CA
Hua et al, 2011	CHF	78 vs 77 (66)	62.7	SJC + AHD vs AHD	8 wk	HAMD, AEs
Lu et al, 2021	CHD	30 (16) vs 30 (12)	69.0 vs 63.0	SJC + AHD vs AHD	8 wk	HAMD

AEs = adverse events;, AHD = antihypertensive drugs, AMI = acute myocardial infarction, CA = cardiac safety, CHD = coronary heart disease, CHF = chronic heart failure, HAMD = Hamilton depression rating scale, n.r. = not reported.

### 3.3. Risk of bias assessment

**Figure 2.**
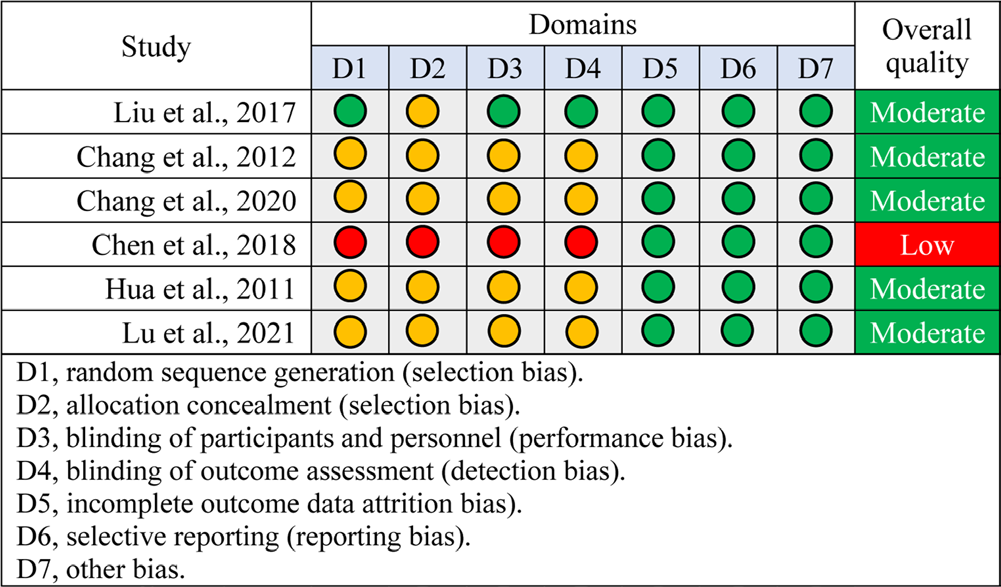
Risk of bias summary of each eligible study. Green, yellow and red circle indicates low, unclear and high risk of bias, respectively.

Among 6 included studies, only 1 study^[29]^ provided a sufficient description of how to generate a random sequence, and 1 study^[27]^ incorrectly conducted randomization. Five studies^[25,26,28–30]^ provided insufficient information about allocation concealment, and 1 study did not conceal allocation.^[27]^ One study^[29]^ adequately blinded participants, personnel, and outcome assessors; however, 1 study^[27]^ did not blind participants, personnel, and outcome assessors. Moreover, the remaining 4 studies^[25,26,28,30]^ did not provide information about these 2 domains. All studies reported the pre-designed outcomes; thus, they did not introduce other biases. Overall, 5 studies had moderate quality, and 1 study had low quality. Details of the risk of bias for all eligible studies are summarized in Figure 2.

### 3.4. Meta-analysis of comparative effect between SGJYC and comparators

**Figure 3.**
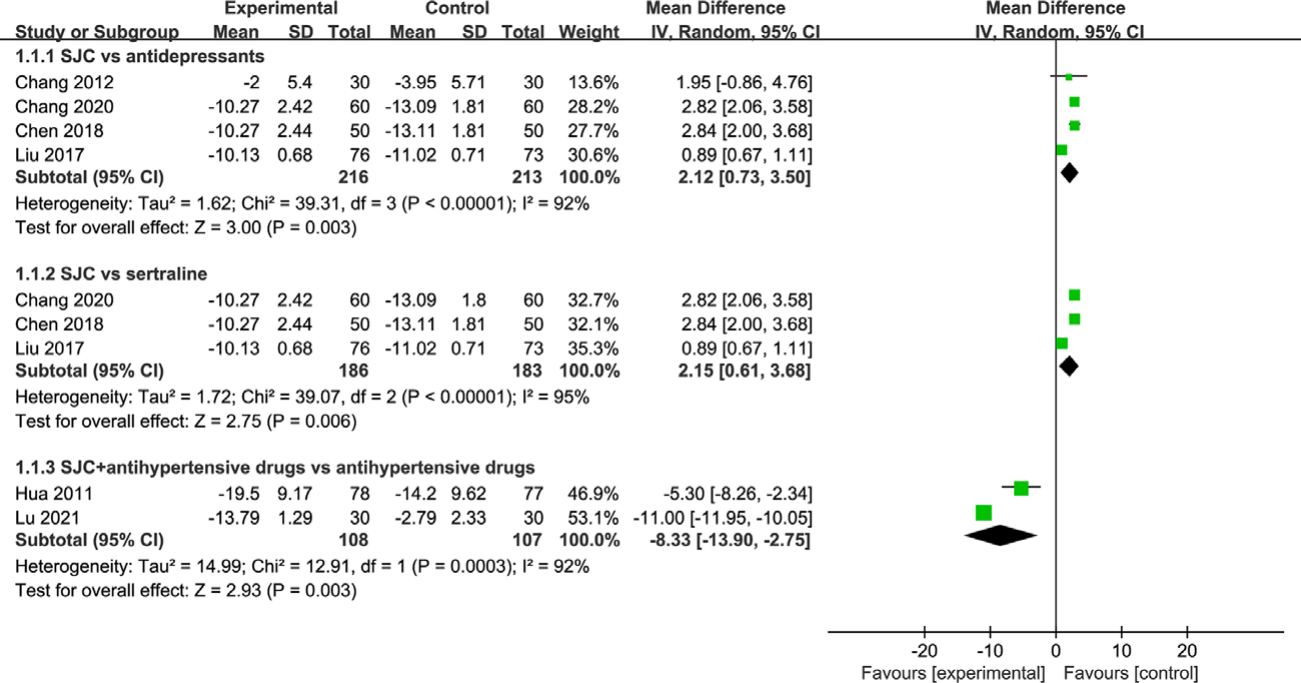
Meta-analysis of anti-depressive effects of different comparisons.

Among 6 eligible studies, 5 regimens were identified, including SGJYC, SGJYC plus antihypertensive drugs (AHD), sertraline, fluoxetine, and AHD. Therefore, we separately estimated the comparative effect of SGJYC-based regimens and other comparators in treating CHD complicated with depression. Meta-analysis indicated a lower depression level in antidepressants (MD, 2.12; 95% CI, 0.73 to 3.50; Z = 3.00, *P* = .003, Fig. 3) or sertraline (MD, 2.15; 95% CI, 0.61 to 3.68; Z = 2.75, *P* < .001, Fig. 3) group compared with SGJYC alone group, with significantly greater changes in HAMD score. However, SGJYC plus AHD was superior to AHD alone in improving depression levels (MD, −8.33, 95% CI, −13.90 to −2.75; Z = 2.93, *P* = .003, Fig. 3).

**Figure 4.**
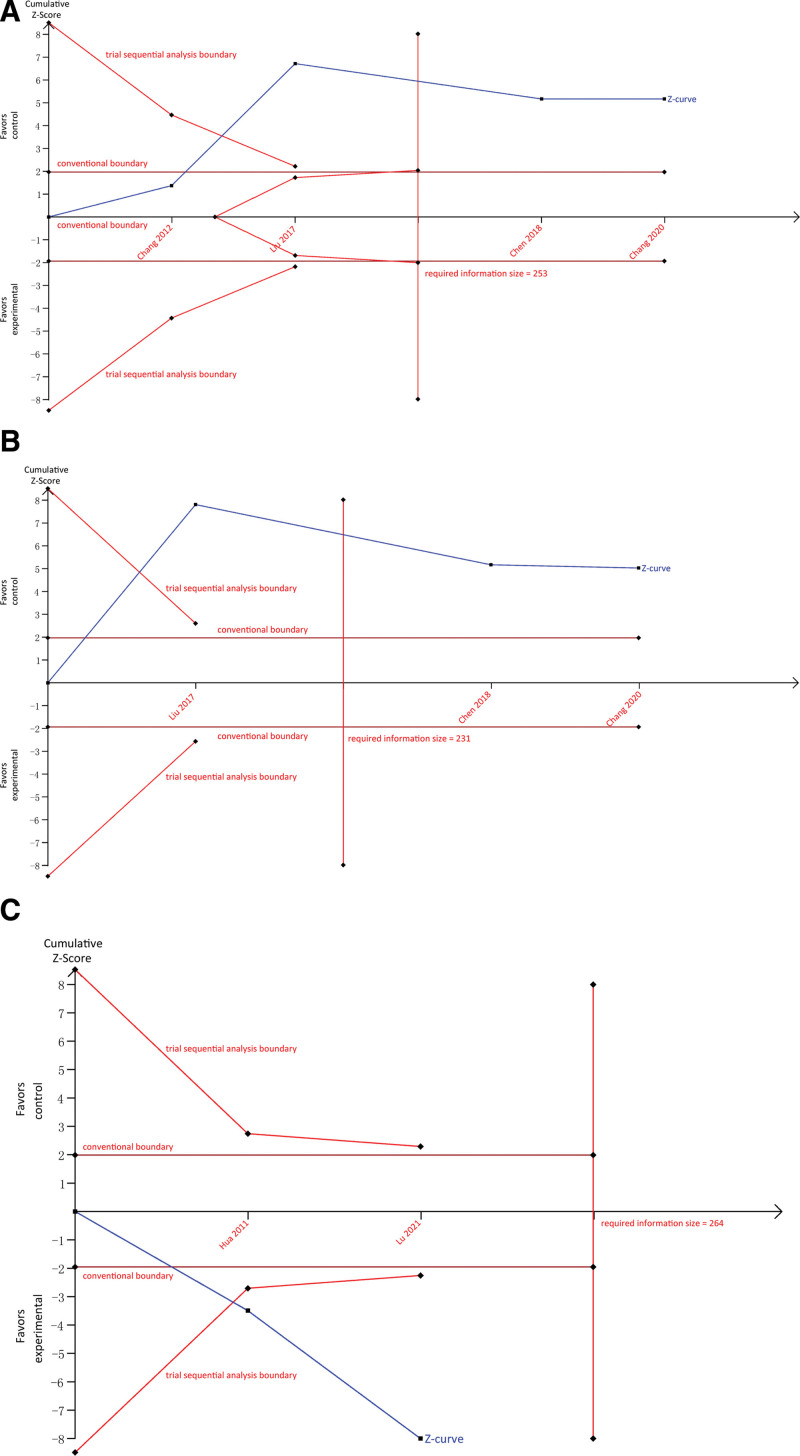
Trial sequential analysis of different comparisons in terms of the anti-depressive effect.

Because only limited eligible studies were included in the present meta-analysis; therefore, we performed trial sequential analysis to examine the robustness of estimates. The required information size of 253 and 231 patients were estimated for the comparisons of SGJYC with antidepressants (Fig. 4A) or sertraline (Fig. 4B), and the accumulated number of patients exceeded the required information sizes. Moreover, the cumulative Z-curves of the 2 comparisons crossed through the trial sequential analysis boundaries, indicating that definitive conclusions have been drawn. For the comparison between SGJYC plus AHD and AHD alone, a required information size of 264 was estimated (Fig. 4C). The cumulative Z-curve crossed through the trial sequential analysis boundary although the accumulated number of patients did not exceed the required information size, also indicating that a definitive conclusion has been drawn. We also calculated estimates based on multiple synthesis methods (i.e., D-L, S-J, and B-T) and the results indicated a robust finding (Table 2).

**Table 2:** Sensitive analysis according to different synthesis methods in meta-analysis.

Synthesis method	HAMD (SJC vs ADs)	HAMD (SJV vs sertraline)	HAMD (SJC + AHD vs AHD)	CA (SJC vs ADs)	CA (SJV vs sertraline)
DerSimonian-Laird (D-L)	2.12 (0.73 to 3.50)	2.15 (0.61 to 3.68)	−8.33 (−13.90 to −2.75)	2.72 (1.07 to 6.94)	3.74 (1.28 to 10.93)
Sidik-Jonkman (S-J)	2.09 (1.08 to 3.09)	2.13 (0.85 to 3.41)	−8.34 (−13.75 to −2.93)	2.65 (0.91 to 7.74)	3.74 (1.28 to 10.94)
Biggerstaff-Tweedie (B-T)	1.39 (0.86 to 1.92)	1.39 (0.84 to 1.93)	−10.23 (−11.84 to −8.62)	2.69 (1.72 to 4.21)	3.74 (2.09 to 6.72)

AHD = antihypertensive drug, CA = cardiac safet, HAMD = Hamilton depression rating scale, SJC = Shugan Jieyu capsule.

### 3.5. Meta-analysis of cardiac safety between SGJYC and sertraline

**Figure 5.**
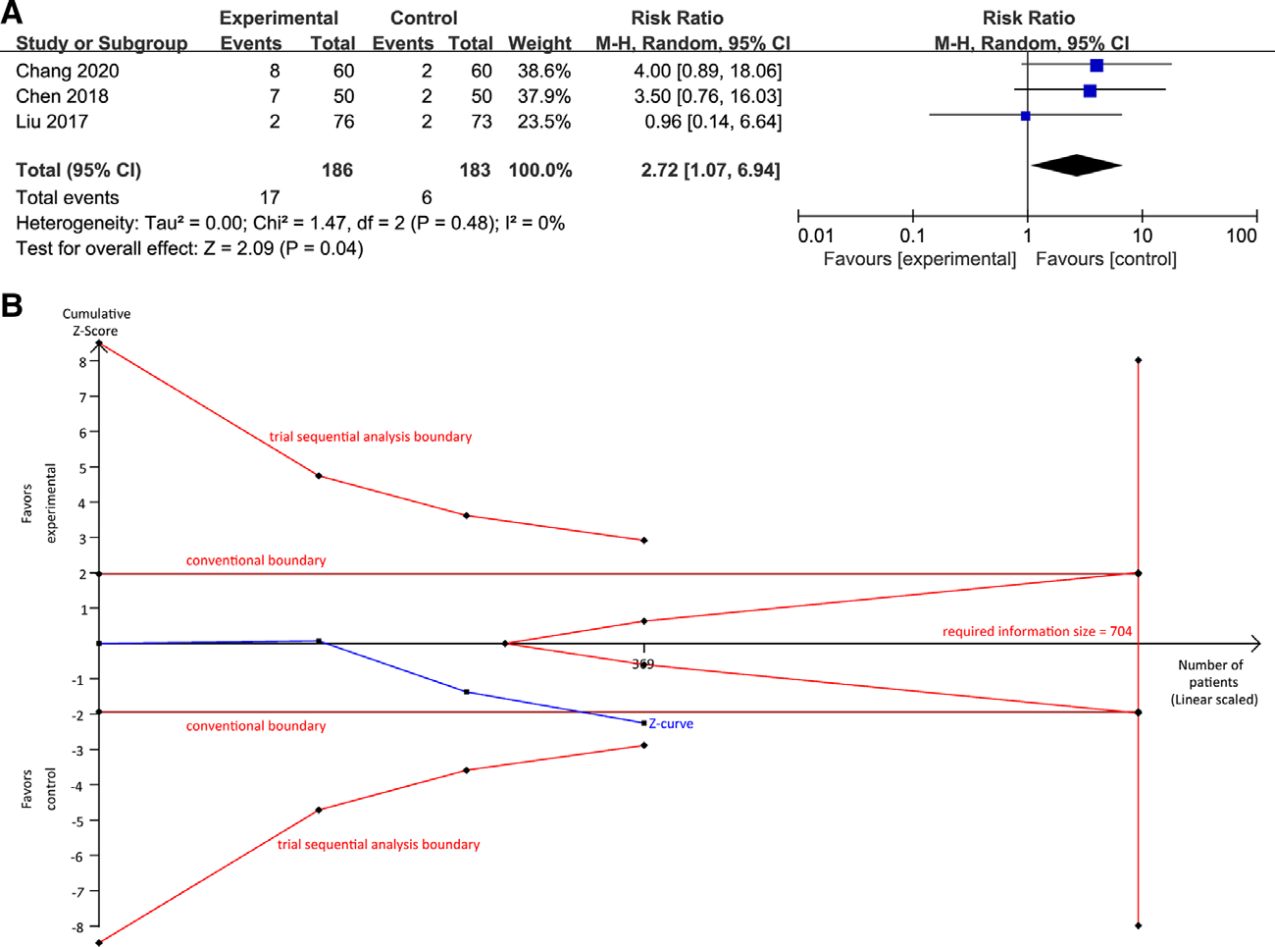
Meta-analysis of cardiac safety between SGJYC and sertraline (A) and trial sequential analysis of the pooled estimate of cardiac safety (B). SGJYC = Shugan Jieyu Capsule.

Among 6 eligible studies, 3 studies that compared SGJYC with sertraline evaluated cardiac safety after the treatment. Meta-analysis suggested that SGJYC had inferior cardiac safety compared with sertraline, with a pooled RR of 2.72 (95% CI, 1.07 to 6.94; Z = 2.09, *P* = .04, Fig. 5A). However, the trial sequential analysis estimated a required information size of 704 (Fig. 5B), and the accumulated number of patients did not exceed it. Meanwhile, the cumulative Z-curve crossed through the conventional boundary but did not cross through trial sequential analysis boundary (Fig. 5B), indicating a false positive finding for this outcome.

**Figure 6.**
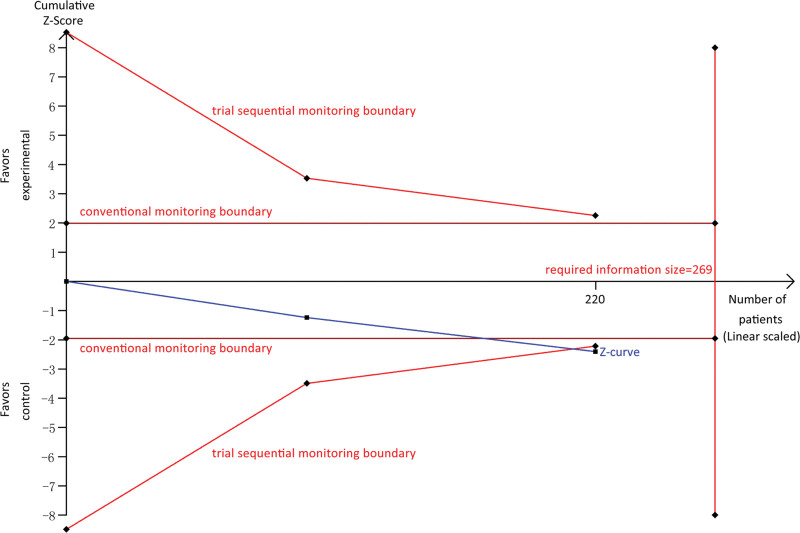
Trial sequential analysis of the pooled estimate of cardiac safety between SGJYC and sertraline among patients with simple C. SGJYC = Shugan Jieyu Capsule.

We further separately investigated the comparative risk of adverse cardiac events among patients with CHD by excluding that study enrolled patients with acute MI. The pooled result indicated a significantly higher risk of cardiac events in the SGJYC group (RR, 3.74; 95% CI, 1.28 to 10.93; Z = 2.42, *P* = .02). Meanwhile, the trial sequential analysis revealed that the cumulative Z-curve crossed through the trial sequential monitoring boundary although the accumulated number of patients did not obtain the required information size (RIS = 269, Fig. 6).

### 3.6. Qualitative summary of adverse events

Among 6 eligible studies, 2 studies reported adverse events after treatment. Liu and colleagues^[29]^ reported that SGJYC was associated with a significantly lower adverse event rate than sertraline. Moreover, another study performed by Hua et al reported comparable adverse events in both SGJYC plus AHD and AHD alone groups and all adverse events did not have a significant impact on clinical outcome.^[28]^ There is a definite relation between CHD and psychosocial factors, especially depressive symptoms.^[11]^ Approximately 55.3% of patients with CHD suffered from different degrees of negative psychology.^[4]^ A previous meta-analysis even suggested a pooled prevalence of 29% for depression among patients with MI.^[43]^ Under this, the concurrent administration of the antidepressants and cardiovascular drugs may improve the prognosis of patients with CHD complicated with depression; however, definite adverse cardiac reactions of most antidepressants have attracted attention.^[16]^ As one kind of Chinese patent medicine, SGJYC has been extensively used to treat various depression in China.^[22]^ However, the anti-depressive effect and safety of SGJYC remain conflicting related to antidepressants. In the present meta-analysis, we demonstrated the anti-depressive effect of SGJYC in patients with CHD complicated with depression because it significantly reduced the HAMD score; however, SGJYC was inferior to antidepressants such as sertraline. More importantly, the administration of SGJYC was associated with a higher risk of adverse cardiac events compared with sertraline among patients with CHD complicated with depression, although there was no significant difference between these 2 regimens among patients with AMI complicated with depression. The clinical medication for anxiety and depression is mainly Western medicine. However, most patients with stable angina pectoris are elderly, and most of them are complicated with other diseases. There are many kinds of drugs. Because of drug interaction, western medicine has poor tolerance in patients with CHD, and can not clearly improve the cardiac prognosis of patients. SGJYC can increase the concentration of monoamine transmitters in the protrusion space by inhibiting the central system reuptake of epinephrine, dopamine, 5-hydroxytryptamine and other neurotransmitters, thus playing an antidepressant role

The active ingredients of the SGJYC contained Acanthopanax and Hypericum perforatum L.^[17]^ Acanthopanax is typically called ciwujia or Siberian ginseng. As a traditional Chinese medicine, it has the property of ameliorating cognitive dysfunction by inhibiting cholinergic blockade.^[44]^ Hypericum perforatum L, also known as St. John wort, has been clinically used to treat depression symptoms and improve cognitive function.^[45]^ Studies suggested the positive effects on mood and short-term verbal memory after using Hypericum perforatum L.^[46]^ Consequently, SGJYC naturally deserves to have anti-depressive effects. It noted that SGJYC has been confirmed to have similar effects but fewer adverse reactions than escitalopram in alleviating depressive symptoms.^[19]^ Moreover, practitioners have also clinically used SGJYC to ameliorate depression and improve cognitive function among patients with PSD.^[47]^ Our study showed that, in anti-depressive effects, there is a significant difference between the SGJYC plus AHD and the AHD alone groups, suggesting SGJYC has clinical benefits in the treatment of mild-to-moderate depression. Certainly, we must acknowledge that the administration of SGJYC alone has an inferior anti-depressive effect than antidepressants, especially sertraline.

## 4. Discussion

Moreover, we also investigated cardiac safety when SGJYC was compared with sertraline. Trial sequential meta-analysis confirmed that SGJYC was associated with an increased risk of adverse cardiac events among patients with CHD. Interestingly, an eligible study reported a similar cardiac safety between SGJYC and sertraline among patients with AMI.^[29]^ However, further studies should be developed to investigate this issue among different populations because no other evidence confirms it.

Our meta-analysis has several strengths as follows: We simultaneously searched Chinese and international databases to retrieve relevant eligible studies. It necessary to search Chinese databases because SGJYC is a Chinese patent drug first approved by the China Food and Drug Administration. We performed a series of subgroup analyses to identify the comparative anti-depressive effects of different comparisons, which were never performed in the other studies. We used trial sequential analysis to eliminate the possibility of false-positive results for quantitative results. We performed sensitive analysis by calculating estimates based on different synthesis methods to further confirm the quantitative results’ robustness.

Several limitations in this meta-analysis must be further interpreted. First and foremost, significant statistical heterogeneity was detected in our meta-analysis because some eligible studies enrolled patients with specific CHD, such as acute MI and CHF, and designed different treatment durations. However, we performed subgroup analysis to eliminate the negative impact of statistical heterogeneity on the estimates as much as possible. Second, we couldn’t investigate the comparative effect and safety of all comparisons among specific patients due to the limited eligible studies. Third, most eligible studies did not report the effect of SGJYC on cardiac functions; therefore, we couldn’t evaluate the cardioprotective effect of SGJYC. In conclusion, the results of this meta-analysis with trial sequential analysis suggest that SGJYC has a significant anti-depressive effect among patients with CHD complicated with depression. Still, its anti-depressive effect and cardiac safety are inferior to antidepressants, especially sertraline. However, only 1 study reported the comparative cardiac safety between SGJYC and sertraline in patients with acute MI; it is therefore imperative to further investigate this result.

## Author contributions

**Conceptualization:** Zhen Liu, Jian Lei.

**Data curation:** Zhen Liu, Chunhua Gu, Jian Lei.

**Formal analysis:** Zhen Liu, Chunhua Gu, Jian Lei.

**Project administration:** Chunhua Gu.

**Writing – original draft:** Zhen Liu, Jian Lei.

## Supplementary Material



## References

[1] DAI Study Group. The prevalence of coronary heart disease in Type 2 diabetic patients in Italy: the DAI study. Diabet Med. 2004;21:738–45. 15209767 10.1111/j.1464-5491.2004.01230.x

[2] O’Neill D Britton A Hannah MK. Association of longitudinal alcohol consumption trajectories with coronary heart disease: a meta-analysis of six cohort studies using individual participant data. BMC Med. 2018;16:124. 30131059 10.1186/s12916-018-1123-6 PMC6103865

[3] Strodl E Kenardy J. A history of heart interventions moderates the relationship between psychological variables and the presence of chest pain in older women with self-reported coronary heart disease. Br J Health Psychol. 2013;18:687–706. 23217000 10.1111/bjhp.12011

[4] Zhang Y Liang Y Huang H. Systematic review and meta-analysis of psychological intervention on patients with coronary heart disease. Ann Palliat Med. 2021;10:8848–57. 34328010 10.21037/apm-21-1623

[5] Rozanski A. Behavioral cardiology: current advances and future directions. J Am Coll Cardiol. 2014;64:100–10. 24998134 10.1016/j.jacc.2014.03.047

[6] Gan Y Gong Y Tong X. Depression and the risk of coronary heart disease: a meta-analysis of prospective cohort studies. BMC Psychiatry. 2014;14:371. 25540022 10.1186/s12888-014-0371-z PMC4336481

[7] Meijer A Conradi HJ Bos EH. Prognostic association of depression following myocardial infarction with mortality and cardiovascular events: a meta-analysis of 25 years of research. Gen Hosp Psychiatry. 2011;33:203–16. 21601716 10.1016/j.genhosppsych.2011.02.007

[8] Hagstrom E Norlund F Stebbins A. Psychosocial stress and major cardiovascular events in patients with stable coronary heart disease. J Intern Med. 2018;283:83–9. 28960596 10.1111/joim.12692

[9] Kuhlmann SL Arolt V Haverkamp W. Prevalence, 12 month prognosis, and clinical management need of depression in coronary heart disease patients: a prospective cohort study. Psychother Psychosom. 2019;88:30001. 10.1159/000501502 31450228

[10] Song X Song J Shao M. Depression predicts the risk of adverse events after percutaneous coronary intervention: a meta-analysis. J Affect Disord. 2020;266:158–64. 32056871 10.1016/j.jad.2020.01.136

[11] Carney RM Freedland KE. Depression and coronary heart disease. Nat Rev Cardiol. 2017;14:145–55. 27853162 10.1038/nrcardio.2016.181

[12] Zhang Y Shuping S. Observation of curative effect of Shugan Jieyu Capsule in the treatment of hypertension, anxiety and depression. Chin J Healthc Nutr. 2018;28:102–3.

[13] Vaccarino V Badimon L Bremner JD. Depression and coronary heart disease: 2018 position paper of the ESC working group on coronary pathophysiology and microcirculation. Eur Heart J. 2020;41:1687–96. 30698764 10.1093/eurheartj/ehy913 PMC10941327

[14] Sweda R Siontis GCM Nikolakopoulou A. Antidepressant treatment in patients following acute coronary syndromes: a systematic review and Bayesian meta-analysis. ESC Heart Fail. 2020;7:3610–20. 32935927 10.1002/ehf2.12861 PMC7754966

[15] Tao J Kong L Fang M. The efficacy of Tuina with herbal ointment for patients with post-stroke depression: study protocol for a randomized controlled trial. Trials. 2021;22:504. 34321056 10.1186/s13063-021-05469-1 PMC8320029

[16] Yekehtaz H Farokhnia M Akhondzadeh S. Cardiovascular considerations in antidepressant therapy: an evidence-based review. J Tehran Heart Cent. 2013;8:169–76. 26005484 PMC4434967

[17] Yang XW. Curative effect of Shugan jieyu capsule on depression with migraine. J Shandong Med Coll. 2015;37:105–7.

[18] Fan M Guo D Tian Y. Efficacy and safety of Shugan Jieyu capsule in the treatment of essential hypertension with insomnia, anxiety or depression: a protocol for systematic review and meta-analysis. Medicine (Baltim). 2021;100:e24856. 10.1097/MD.0000000000024856 PMC7909162 33663107

[19] Sun XY Chen AQ Xu XW. Randomized, double blind, placebo-controlled trial of Shuganjieyu capsule in the treatment of mild or moderate depression [in Chinese]. Chin J New Drugs. 2009;18:413–6.

[20] Zhang M Bai X. Shugan Jieyu capsule in post-stroke depression treatment: from molecules to systems. Front Pharmacol. 2022;13:821270–821270. 35140618 10.3389/fphar.2022.821270 PMC8818889

[21] Fu J Zhang Y Wu R. Shuganjieyu capsule increases neurotrophic factor expression in a rat model of depression. Neural Regen Res. 2014;9:489–97. 25206843 10.4103/1673-5374.130067 PMC4153504

[22] Pu ZP Xia JM Xie W. Exploring the clinical characters of Shugan Jieyu capsule through text mining. Zhongguo Zhong Yao Za Zhi. 2017;42:3430–3. 29192458 10.19540/j.cnki.cjcmm.20170710.002

[23] Wu T Yue T Yang P. Notable efficacy of Shugan Jieyu capsule in treating adult with post-stroke depression: a PRISMA-compliant meta-analysis of randomized controlled trials. J Ethnopharmacol. 2022;294:115367. 35562090 10.1016/j.jep.2022.115367

[24] Yao G Zhang X Li J. Improving depressive symptoms of post-stroke depression using the Shugan Jieyu capsule: a resting-state functional magnetic resonance imaging study. Front Neurol. 2022;13:860290. 35493835 10.3389/fneur.2022.860290 PMC9047823

[25] Chang J Yuan HM Yang WH. Antidepressant treatment of elderly heart failure patients complicated with depressed in traditional Chinese medicine and western medicine [in Chinese]. J Psychiatry. 2012;25:179–81.

[26] Chang N Zhao LL Hu J. Effect of sertraline on negative emotion and cardiovascular adverse events in patients with coronary heart disease complicated with depression [in Chinese]. Chin Commun Doctors. 2020;36:58–9.

[27] Chen ML Zhu XL He CH. Effect of sertraline on negative emotion and adverse cardiovascular events in patients with coronary heart disease complicated with depression [in Chinese]. Chin J Postgrad Med. 2018;41:605–8.

[28] Hua XP Chen PY Yang Y. Effect of Shugan Jieyu capsule in the treatment of patients with chronic heart failure complicated with depression [in Chinese]. Chin J Gerontol. 2011;31:3502–4.

[29] Liu W Qin J. Clinical efficacy and safety of the Shugan Jieyu capsule in patients with acute myocardial infarction and depression. Int J Psychiatry Med. 2016;51:534–43. 28629293 10.1177/0091217417696740

[30] Lu XZ Liu L Liu ZK. Effect of Shugan jieyu capsule for the treatment of patients with coronary heart disease complicated with depression [in Chinese]. Sci Regimen. 2021;24:220.

[31] Higgins JPT Thomas J Chandler J. Cochrane Handbook for Systematic Reviews of Interventions version 6.2 (updated February 2021). Cochrane, 2021. Available at: www.training.cochrane.org/handbook.

[32] Page MJ McKenzie JE Bossuyt PM. The PRISMA 2020 statement: an updated guideline for reporting systematic reviews. BMJ. 2021;372:n71. 33782057 10.1136/bmj.n71 PMC8005924

[33] Page MJ Moher D Bossuyt PM. PRISMA 2020 explanation and elaboration: updated guidance and exemplars for reporting systematic reviews. BMJ. 2021;372:n160. 33781993 10.1136/bmj.n160 PMC8005925

[34] Tanon AA Champagne F Contandriopoulos AP. Patient safety and systematic reviews: finding papers indexed in MEDLINE, EMBASE and CINAHL. Qual Saf Health Care. 2010;19:452–61. 20457733 10.1136/qshc.2008.031401

[35] Wan X Wang W Liu J. Estimating the sample mean and standard deviation from the sample size, median, range and/or interquartile range. BMC Med Res Methodol. 2014;14:135. 25524443 10.1186/1471-2288-14-135 PMC4383202

[36] Higgins JP Altman DG Gotzsche PC. The Cochrane Collaboration’s tool for assessing risk of bias in randomised trials. BMJ. 2011;343:d5928. 22008217 10.1136/bmj.d5928 PMC3196245

[37] DerSimonian R Laird N. Meta-analysis in clinical trials. Control Clin Trials. 1986;7:177–88. 3802833 10.1016/0197-2456(86)90046-2

[38] Röver C Knapp G Friede T. Hartung-Knapp-Sidik-Jonkman approach and its modification for random-effects meta-analysis with few studies. BMC Med Res Methodol. 2015;15:99. 26573817 10.1186/s12874-015-0091-1 PMC4647507

[39] Biggerstaff BJ Tweedie RL. Incorporating variability in estimates of heterogeneity in the random effects model in meta-analysis. Stat Med. 1997;16:753–68. 9131763 10.1002/(sici)1097-0258(19970415)16:7<753::aid-sim494>3.0.co;2-g

[40] Wetterslev J Thorlund K Brok J. Trial sequential analysis may establish when firm evidence is reached in cumulative meta-analysis. J Clin Epidemiol. 2008;61:64–75. 18083463 10.1016/j.jclinepi.2007.03.013

[41] Brok J Thorlund K Gluud C. Trial sequential analysis reveals insufficient information size and potentially false positive results in many meta-analyses. J Clin Epidemiol. 2008;61:763–9. 18411040 10.1016/j.jclinepi.2007.10.007

[42] Thorlund K Engstrøm J Wetterslev J. User Manual for Trial Sequential Analysis (TSA) [pdf]. 2nd ed. Copenhagen: Copenhagen Trial Unit, 1–119. Downloadable from ctu.dk/tsa [accessed date 09, 17, 2021]. [Online]. 2017.

[43] Feng L Li L Liu W. Prevalence of depression in myocardial infarction: a PRISMA-compliant meta-analysis. Medicine (Baltim). 2019;98:e14596. 10.1097/MD.0000000000014596 PMC6407970 30813183

[44] de Blasio F de Blasio F Miracco Berlingieri G. Evaluation of body composition in COPD patients using multifrequency bioelectrical impedance analysis. Int J Chron Obstruct Pulmon Dis. 2016;11:2419–26. 27757027 10.2147/COPD.S110364 PMC5053371

[45] Avila C Whitten D Evans S. The safety of St John’s wort (Hypericum perforatum) in pregnancy and lactation: a systematic review of rodent studies. Phytother Res. 2018;32:1488–500. 29708295 10.1002/ptr.6099

[46] Yechiam E Ben-Eliezer D Ashby NJS. The acute effect of Hypericum perforatum on short-term memory in healthy adults. Psychopharmacology (Berl). 2019;236:613–23. 30382352 10.1007/s00213-018-5088-0

[47] Yi F Li J Rong JK. Shuganjieyu capsule alone or in combination with other antidepressants for post-stroke depression:a Meta-analysis. Chin J Cerebrovasc Dis. 2018;15:140–7.

