# Supplementary material for: Meta-analysis of Shugan Jieyu Capsule for depression in patients with coronary heart disease
Source: Medicine (Baltimore). 2023 Aug 25;102(34):e34685. doi: 10.1097/MD.0000000000034685 (PMC10470748; doi:10.1097/MD.0000000000034685)
Supplement: Supplementary file 1 [file medi-102-e34685-s001.pdf]

Table S1 China National Knowledgement Infrastructur，CNKI.

China National Knowledgement Infrastructur，CNKI

Search Scope: Total Database (Subject: Soothing Liver and Relieving Depression) AND ((Subject: Coronary Heart Disease + Heart Failure + Myocardial Infarction + Myocardial Infarction + Myocardial Infarction)) AND ((Subject: Anxiety + Depression)) AND ((Full Text: Random))

WANFANG data

Search expression: Subject: (Soothing the liver and relieving depression) and Subject: (heart failure or myocardial infarction or myocardial infarction or myocardial infarction or coronary heart disease) and subject: (anxiety or depression) and all: (random)

Chinese Biomedical Literature database，CBM or Sinomed

"Random"[all fields] AND "soothe liver and relieve depression"[common fields] AND ((("anxiety"[common fields] OR "anxiety"[subject terms]) OR ("depression"[common fields] OR "depression Symptoms"[common fields] OR "depression"[subject terms])) AND (("myocardial infarction"[common fields] OR "heart attack"[common fields] OR "myocardial infarction"[common fields] OR "myocardial infarction "[Subject word]) OR ("Myocardial infarction"[Common field] OR "Myocardial infarction"[Common field] OR "Heart attack"[Common field] OR "Myocardial infarction"[Subject word]) OR "Myocardial infarction" [Common Fields] OR ("Coronary Heart Disease"[Common Fields] OR "Coronary Artery Heart Disease"[Common Fields] OR "Coronary Heart Disease"[Subject Word])))

Cochrane Central Register of Controlled Trials via OVID

1 (Coronary Diseases or Coronary Heart Disease or Coronary Heart Diseases or Myocardial Infarctions or Cardiovascular Stroke or Cardiovascular Strokes or Myocardial Infarct or Myocardial Infarcts or Heart Attack or Heart Attacks).af.
2 exp Coronary Disease/
3 exp Myocardial Infarction/
4 1 or 2 or 3
5 (shuganjieyu or shugan jieyu or shugan-jieyu or shu-gan-jie-yu).af.
6 (anxiety or depression).af.
7 exp Anxiety/
8 exp Depression/
9 6 or 7 or 8
10 exp Random Allocation/ or random.mp.
11 randomized controlled trial.mp. or exp Randomized Controlled Trial/
12 10 or 11
13 4 and 5 and 9 and 12

MDELINE via PubMed

7 (((("Myocardial Infarction"[Mesh]) OR "Coronary Disease"[Mesh]) OR (((((((((Coronary Diseases[Title/Abstract]) OR (Coronary Heart Disease[Title/Abstract])) OR (Coronary Heart Diseases[Title/Abstract])) OR (Myocardial Infarctions[Title/Abstract])) OR (Cardiovascular Stroke[Title/Abstract])) OR (Cardiovascular Strokes[Title/Abstract])) OR (Myocardial Infarct[Title/Abstract])) OR (Myocardial Infarcts[Title/Abstract])) OR (Heart Attack[Title/Abstract])) 6 (Heart Attacks[Title/Abstract]))) AND ((((shugan jieyu[Title/Abstract]) OR (shuganjieyu[Title/Abstract])) OR (shugan-jeiyu[Title/Abstract])) OR (shu-gan-jie-yu[Title/Abstract]))) AND (random*)

5 random*

4 (((shugan jieyu[Title/Abstract]) OR (shuganjieyu[Title/Abstract])) OR (shugan-jeiyu[Title/Abstract])) OR (shu-gan-jie-yu[Title/Abstract])

3 (("Myocardial Infarction"[Mesh]) OR "Coronary Disease"[Mesh]) OR (((((((((Coronary Diseases[Title/Abstract]) OR (Coronary Heart Disease[Title/Abstract])) OR (Coronary Heart Diseases[Title/Abstract])) OR (Myocardial Infarctions[Title/Abstract])) OR (Cardiovascular Stroke[Title/Abstract])) OR (Cardiovascular Strokes[Title/Abstract])) OR (Myocardial Infarct[Title/Abstract])) OR (Myocardial Infarcts[Title/Abstract])) OR (Heart Attack[Title/Abstract])) OR (Heart Attacks[Title/Abstract]))

2 (((((((((Coronary Diseases[Title/Abstract]) OR (Coronary Heart Disease[Title/Abstract])) OR (Coronary Heart Diseases[Title/Abstract])) OR (Myocardial Infarctions[Title/Abstract])) OR (Cardiovascular Stroke[Title/Abstract])) OR (Cardiovascular Strokes[Title/Abstract])) OR (Myocardial Infarct[Title/Abstract])) OR (Myocardial Infarcts[Title/Abstract])) OR (Heart Attack[Title/Abstract])) OR (Heart Attacks[Title/Abstract])

1 ("Myocardial Infarction"[Mesh]) OR "Coronary Disease"[Mesh]
